# Preparation of Alumina-Sphere-Supported Potassium Chabazite Zeolite Membrane with Excellent Potassium Extraction Performance at Room Temperature

**DOI:** 10.3390/membranes12060604

**Published:** 2022-06-10

**Authors:** Jie Ouyang, Heng Wei, Jin Hou

**Affiliations:** College of Chemistry and Chemical Engineering, Ocean University of China, Qingdao 266100, China; jhhxhg@163.com (J.O.); bhqing266@163.com (H.W.)

**Keywords:** KCHA zeolite membrane, potassium extraction, seawater, sea bittern

## Abstract

In this paper, a potassium chabazite (KCHA) zeolite membrane was prepared by coating KCHA zeolite on the surface of a porous alumina sphere. The performance of the KCHA zeolite membrane in extracting potassium from seawater and sea bittern at room temperature was studied in detail. The XRD results show that the prepared KCHA zeolite was a KCHA membrane. The EDS test indicated that the potassium content of the KCHA zeolite membrane reached a value of 18.33 wt.%. The morphology of the KCHA zeolite grown on the surface of the alumina sphere was similar to a sphere, and it had good symmetry. The potassium ion-exchange capacities of the KCHA zeolite membrane reached 32 mg/g in seawater and 77 mg/g in sea bittern at room temperature. Ion exchange between the ammonium ions and potassium ions in the KCHA zeolite membrane could be completed in a short time at room temperature. The KCHA zeolite membrane was proven to have good reusability in seawater and sea bittern. The selective ion-exchange mechanism of the KCHA zeolite membrane was controlled by a specific K^+^ ion memory.

## 1. Introduction

Potassium is an element essential to maintain the growth of plants. Among the many plant nutrients, potassium plays an especially critical role in all living organisms [1]. About ninety percent of potassium is used for the production of fertilizers [2]. There is a lack of potassium resources on land, but it can be found in high amounts in seawater and sea bittern [3,4]. About 550 trillion tons of potassium exists in seawater. China currently produces 20 million cubic meters of sea bittern per year, which is a by-product of salt production from seawater. The concentrations of various chemical elements in sea bittern are significantly higher in comparison with those in seawater. The concentration ratio of potassium to sodium is markedly higher because of the increased potassium content, which greatly reduces the difficulty of separating potassium from sodium. Therefore, abundant sea bittern resources are excellent materials for potassium extraction. As a result, extracting potassium from seawater and sea bittern has attracted the extensive attention of researchers [5,6,7,8,9].

Zeolites are regarded as important ion-exchange materials for potassium extraction and are widely used in selective separation because of their high ion-exchange capacity and specific surface area [10,11,12,13]. Therefore, potassium extraction using zeolites has attracted research interest. Merlinoite, phillipsite, zeolite P, mordenite, and clinoptilolite have been used for potassium extraction [14,15,16,17,18]. At present, chabazite zeolite or chabazite zeolite membranes are mostly used for gas adsorption, dehydration, catalysis, and purification [19,20,21,22,23]. However, to date, there has been no report on the extraction of potassium by chabazite zeolite or chabazite zeolite membranes.

At present, zeolites usually need to be granulated in practical applications, which causes a reduction in purity and in the potassium extraction performance of zeolite. In addition, the regeneration of zeolite needs a high temperature and a long time, resulting in the high cost of potassium extraction. In order to solve the first problem, in this paper, coating technology was adopted to maintain the high exchange performance of zeolite, which is more feasible. In order to solve the second problem, KCHA zeolite membrane coated alumina microspheres with rapid adsorption and desorption for K^+^ was studied in detail at room temperature. In this work, a KCHA zeolite membrane was prepared and characterized using X-ray diffraction (XRD), energy dispersive X-ray spectroscopy (EDS), and scanning electron microscopy (SEM). Thus, the performance of the KCHA zeolite membrane for extracting potassium from seawater and sea bittern were studied for the first time.

## 2. Materials and Methods

### 2.1. Materials

Chemical reagents, including potassium hydroxide (KOH), sodium aluminate (NaAlO_2_), and ammonium chloride (NH_4_Cl), were purchased from Shanghai Sinopharm Chemical Reagent Co., Ltd. Soluble silicate (SiO_2_, 27.8 wt.%) was purchased from Qingdao, China. A porous alumina sphere (Al_2_O_3_, Φ3–4 mm) was purchased from Zhengzhou, China. Seawater and sea bittern were prepared manually (see Table 1 for main cation content).

### 2.2. Preparation of KCHA Zeolite Membrane

The synthesis solution was prepared by dissolving 27.6 g of soluble silicate, 30.0 g of potassium hydroxide, and 2.60 g of sodium aluminate in deionized water at room temperature. The resulting solution was evenly stirred for 12 h at room temperature, poured slowly into Teflon-lined stainless steel reaction kettles until 16.00 g of the alumina sphere inside was completely immersed, and treated for 24 h at 120 °C. After hydrothermal treatment, the porous alumina-sphere-supported KCHA zeolite membrane was collected by filtration, washed with deionized water, and dried at 100 °C overnight.

### 2.3. Modification of KCHA Zeolite Membrane

**Figure 1:**
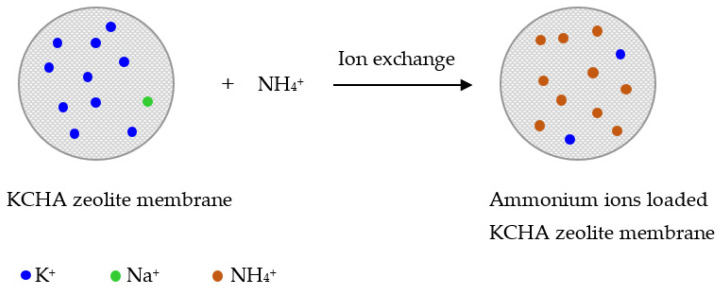
Diagram for modification of KCHA zeolite membrane.

For potassium ion exchange between K^+^ ions in the KCHA zeolite membrane and ammonium ions, the alumina-sphere-supported KCHA zeolite membrane and NH_4_Cl were added to deionized water. The suspension was then continuously stirred for 5 min at room temperature. The ammonium-ion-loaded KCHA zeolite membrane was obtained by filtration, washed with deionized water, and dried at 100 °C overnight. A reaction diagram is shown in Figure 1.

### 2.4. Potassium Extraction and Removal Process of KCHA Zeolite Membrane

**Figure 2:**
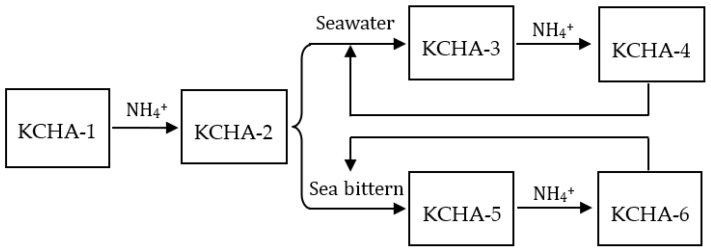
Flow diagram for potassium extraction and removal process.

In order to study the potassium extraction and removal performance of the KCHA zeolite membrane, the following experiments were carried out: The KCHA zeolite membrane (labeled as KCHA-1) was modified with ammonium ions to obtain an ammonium-ion-loaded KCHA zeolite membrane labeled as KCHA-2. The KCHA zeolite membrane labeled as KCHA-3 was recovered after KCHA-2 was added to 500 mL of artificial seawater to absorb potassium for 10 min at room temperature. KCHA-3 was modified again with ammonium ions to obtain an ammonium-ion-loaded KCHA zeolite membrane labeled as KCHA-4. When KCHA-2 was added to 25 mL of artificial sea bittern to absorb potassium for 10 min at room temperature, a KCHA zeolite membrane labeled as KCHA-5 was obtained. KCHA-5 was modified again with ammonium ions to obtain an ammonium-ion-loaded KCHA zeolite membrane labeled as KCHA-6. A flow diagram of the potassium extraction and removal process is shown in Figure 2.

### 2.5. Characterization

The structural feature of KCHA zeolite was evaluated using XRD (Bruker D8-Advance, Karlsruhe, Germany) with λ = 1.5418 Å Cu K_α_ radiation. The surface morphology of the KCHA zeolite membrane was examined using SEM (S4800, Tokyo, Japan), and elemental analysis was performed using EDS (E-max, Tokyo, Japan).

## 3. Results and Discussion

### 3.1. Characterization of KCHA Zeolite Membrane

**Figure 3:**
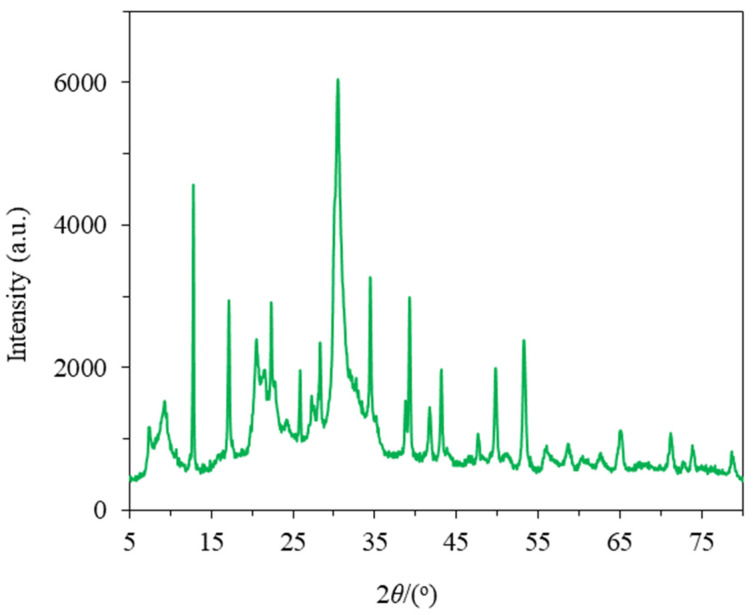
XRD pattern of KCHA zeolite membrane.

The XRD pattern of the KCHA zeolite powder scrapped from the surface of the prepared KCHA zeolite membrane is exhibited in Figure 3. The main diffraction peaks appeared at 12.86°, 22.38°, 30.53°, 34.49°, 39.29°, and 53.26°, which correspond to the characteristic peaks reported for KCHA zeolite [24]. The results indicate that the synthetic membrane was a KCHA zeolite membrane.

**Figure 4:**
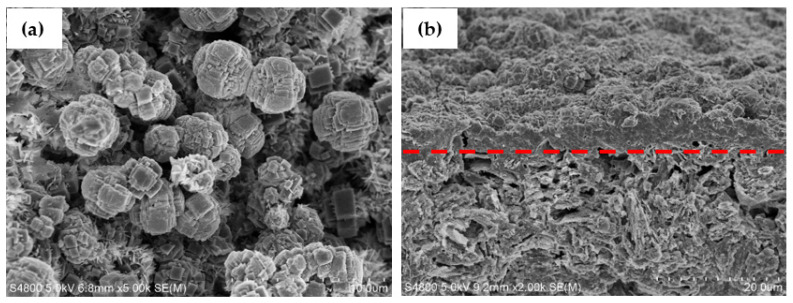
Surface (**a**) and cross-section (**b**) SEM images of KCHA zeolite membrane.

Micrographs of the KCHA zeolite membrane were obtained using SEM. The surface and cross-section SEM photographs are shown in Figure 4a,b. The morphology of the KCHA zeolite grown on the surface of the spherical alumina was similar to a sphere, and it had good symmetry as can be seen in the surface SEM image in Figure 4a. According to the pore size, there was an obvious boundary between the porous alumina sphere and the synthetic KCHA zeolite membrane, as revealed in the cross-section SEM image in Figure 4b. The thickness of the KCHA zeolite membrane was about 5–6 μm.

**Figure 5:**
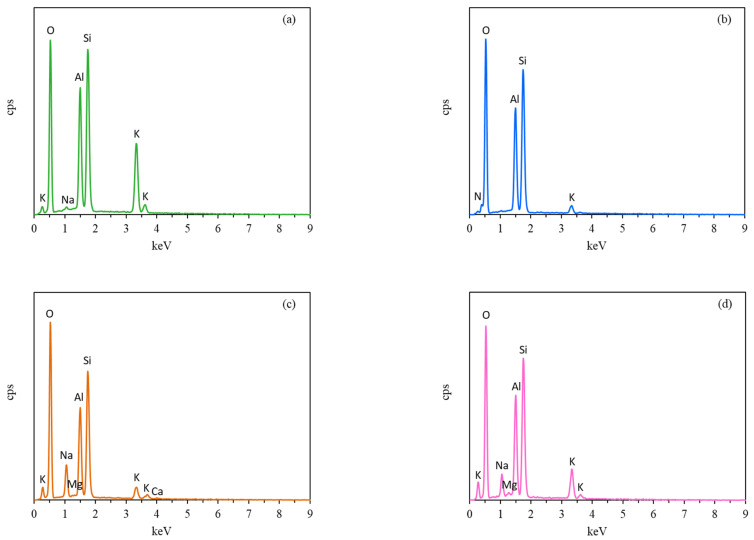
EDS patterns of the synthetic zeolite membranes: (**a**) unmodified KCHA zeolite membrane; (**b**) the ammonium-ion-loaded KCHA zeolite membrane; (**c**) the recovered KCHA zeolite membrane after absorbing potassium from seawater; (**d**) the recovered KCHA zeolite membrane after absorbing potassium from sea bittern.

The elemental components of the synthetic zeolite membranes were determined using EDS, and the results are shown in Figure 5a–d. Figure 5a shows the EDS of the synthetic KCHA zeolite membrane, which was mainly composed of elements K, Si, Al, O, and a very small amount of Na. The KCHA zeolite membrane was modified with ammonium ions to obtain the ammonium-ion-loaded KCHA zeolite membrane, which was mainly composed of elements N, Si, Al, O, and a small amount of K as shown in Figure 5b. Therefore, potassium ions were almost replaced by ammonium ions. The KCHA zeolite membrane was recovered after the ammonium-ion-loaded KCHA zeolite membrane was added to seawater or sea bittern to absorb potassium. As shown in Figure 5c, the EDS result of the recovered KCHA zeolite membrane after absorbing potassium from seawater indicates that the main cations, such as potassium, sodium, and a small amount of calcium and magnesium, appeared. As shown in Figure 5d, the EDS result of the recovered KCHA zeolite membrane after absorbing potassium from sea bittern indicates that the main cations, such as potassium, sodium, and a small amount of calcium, appeared. Therefore, the main cations in seawater and sea bittern, such as potassium and sodium ions, entered the pores of the zeolite membrane to replace the ammonium ions. The KCHA zeolite membrane can be used for further research on potassium extraction from seawater and sea bittern.

### 3.2. Potassium Extraction Performance of KCHA Zeolite Membrane

The weight %s of the elements (N, K, Na, Mg, and Ca) in KCHA-1, KCHA-2, KCHA-3, KCHA-4, KCHA-5, and KCHA-6 during the potassium extraction and removal processes as shown in Figure 2 were measured using EDS, and the results are shown in Table 2. KCHA-1, KCHA-3, and KCHA-5 were potassium-ion-loaded chabazite zeolites. KCHA-2, KCHA-4, and KCHA-6 were ammonium-ion-loaded chabazite zeolites.

The content of potassium in KCHA-2 was very low, indicating that potassium was mostly replaced by ammonium ions. After KCHA-3 and KCHA-5 were modified again with ammonium ions, nitrogen comprised a large proportion of KCHA-4 and KCHA-6. The main ions adsorbed from seawater were potassium and sodium, with very low amounts of magnesium and calcium found in KCHA-3. The main ions adsorbed from sea bittern were also potassium and sodium for KCHA-5. Because the concentration ratio of potassium and sodium in sea bittern is higher than that in seawater, the amount of potassium adsorbed from sea bittern by the ammonium-ion-loaded KCHA zeolite membrane was significantly higher than that of sodium. In sea bittern, the difficulty of separating potassium from sodium was reduced. The potassium ion-exchange capacities of the KCHA zeolite membrane were 32 mg/g in seawater and 77 mg/g in sea bittern. The potassium ion-exchange capacity in sea bittern was more than twice that in seawater. It was basically consistent with the potassium ion-exchange capacity of merlinoite previously reported by us, but it was much higher than that of P zeolite reported by Cao [16]. The concentration ratio of potassium to sodium in sea bittern was obviously higher than that in seawater. Thus, the difficulty of separating potassium from sodium was reduced in sea bittern.

The potassium ion-exchange capacities of the KCHA zeolite membrane were calculated using the formula given below:

$$Q_{K}=\frac{m_{1}\omega_{1}-m_{2}\omega_{2}}{m}$$

where *Q*_K_ (mg/g) is the potassium ion-exchange capacity of the KCHA zeolite membrane, *m* (g) is the mass of the KCHA zeolite membrane, *m*_1_ (mg) is the mass of KCHA-3 (KCHA-5), *ω*_1_ is the weight % of potassium in KCHA-3 (KCHA-5), *m*_2_ (mg) is the mass of KCHA-4 (KCHA-6), and *ω*_2_ is the weight % of potassium in KCHA-4 (KCHA-6).

### 3.3. Reusability of KCHA Zeolite Membrane

**Figure 6:**
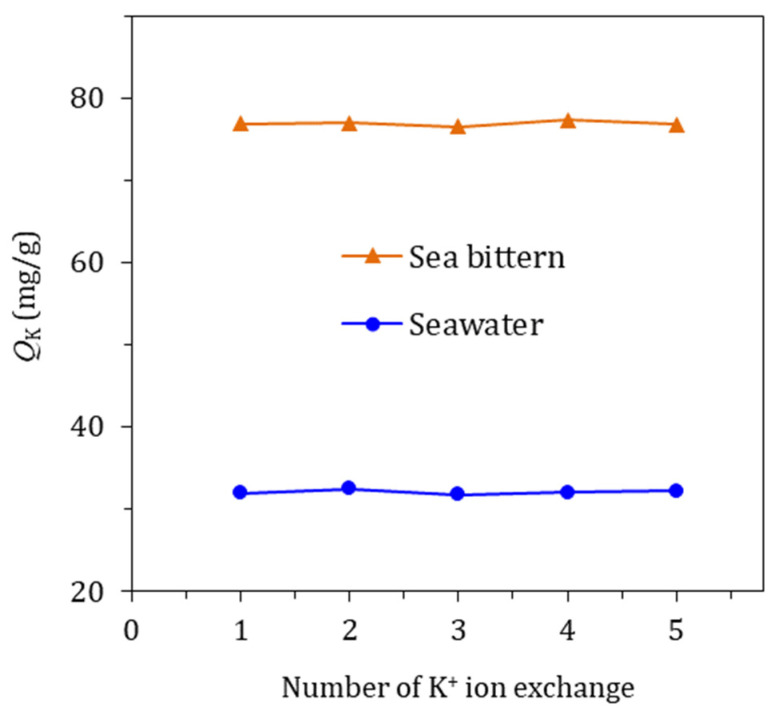
Reusability of KCHA zeolite membrane.

The KCHA zeolite membrane was first modified with ammonium ions and then added to seawater or sea bittern to extract potassium. This was repeated five times. Five *Q*_K_ of the KCHA zeolite membrane is shown in Figure 6. The results indicate the *Q*_K_ of the KCHA zeolite membrane did not change noticeably. It was found that the KCHA zeolite membrane possessed great reusability, and it can be sustainably used to extract potassium from seawater and sea bittern.

### 3.4. Selective Ion-Exchange Mechanism of KCHA Zeolite Membrane

The selective ion-exchange mechanism of the KCHA zeolite membrane is proposed as follows: Potassium hydroxide was used for the preparation of the KCHA zeolite membrane. Potassium hydroxide not only provided alkalinity but also extra K^+^ ions, which played a major part in preparing the KCHA zeolite membrane. Potassium ions were first introduced into the KCHA zeolite membrane as target ions. As a result, the ammonium-ion-loaded KCHA zeolite membrane prepared from the modification of the KCHA zeolite membrane had a particular ability to remember K^+^ ions. The diameter of sodium ions is similar to that of potassium ions, causing sodium ions to be competitive ions with respect to potassium ions when replacing ammonium ions of the ammonium-ion-loaded KCHA zeolite membrane. Sodium ions are major disruptive ions for extracting potassium from seawater and sea bittern [25,26].

## 4. Conclusions

The potassium extraction performance of KCHA zeolite membranes was studied for the first time. A KCHA zeolite membrane with a high potassium content of 18.33 wt.% was successfully prepared. The *Q*_K_ of the KCHA zeolite membrane reached 32 mg/g in seawater and 77 mg/g in sea bittern. The potassium ion-exchange capacity in sea bittern was more than twice that in seawater. The concentration ratio of potassium to sodium in sea bittern was obviously higher than that in seawater, so it was relatively easy to separate potassium and sodium from sea bittern. Potassium ions in the KCHA zeolite membrane were exchanged with ammonium ions in a short time at room temperature. The KCHA zeolite membrane presented fine reusability, and it can be sustainably used to extract potassium from seawater and sea bittern. The KCHA zeolite membrane had the ability of K^+^ ion memory, allowing for the selective identification of K^+^ ions. The alumina-sphere-supported KCHA zeolite membrane has the characteristics of rapid potassium extraction and removal at room temperature, and it is expected to be widely used and popularized in the future.

## Figures and Tables

**Table 1:** Concentration of major cations in seawater and sea bittern.

Cation	Seawater	Sea Bittern
K^+^ (mg∙mL^−1^)	0.38	10.67
Na^+^ (mg∙mL^−1^)	10.62	39.71
Mg^2+^ (mg∙mL^−1^)	1.28	57.59
Ca^2+^ (mg∙mL^−1^)	0.40	--

**Table 2:** Weight %s of elements N, K, Na, Mg, and Ca as determined using EDS.

	Sample	KCHA-1	KCHA-2	KCHA-3	KCHA-4	KCHA-5	KCHA-6
Element							
N (wt.%)		--	7.54	--	7.64	--	7.96
K (wt.%)		18.33	2.75	4.25	1.06	9.46	1.77
Na (wt.%)		0.31	--	5.57	0.24	3.46	0.16
Mg (wt.%)		--	--	0.11	--	0.36	
Ca (wt.%)		--	--	1.63	--		

## Data Availability

Data are available upon request.
